# Circ_0008532 promotes bladder cancer progression by regulation of the miR-155-5p/miR-330-5p/MTGR1 axis

**DOI:** 10.1186/s13046-020-01592-0

**Published:** 2020-05-27

**Authors:** Liang Chen, Xiong Yang, Jun Zhao, Ming Xiong, Raya Almaraihah, Zhaohui Chen, Teng Hou

**Affiliations:** Department of Urology, Union Hospital, Tongji Medical College, Huazhong University of Science and Technology, Wuhan, 430022 HB China

**Keywords:** Circ_0008532, Bladder cancer, MTGR1, Notch

## Abstract

**Background:**

Circular RNAs (circRNAs) have been associated with bladder cancer (BC), but the specific underlying molecular mechanism of their association with BC development has not been fully explored.

**Methods:**

Levels of Circ_0008532, MTGR1 and miR-155-5p/miR-330-5p in bladder cancer cell lines and tissues were determined with quantitative real-time PCR and western blotting assays. In vitro and in vivo assays were performed to investigate the function of circ_0008532 in tumorigenesis in bladder cancer cells. The relationships of Circ_0008532, MTGR1 and miR-155-5p/miR-330-5p were predicted using bioinformatic tools and verified by RNA-FISH, RIP and luciferase assays. The effects of circ_0008532 on the Notch signaling pathway were determined by GSEA analysis and western blotting assay.

**Results:**

We found that circ_0008532 is upregulated in BC cell lines and tissues. Moreover, overexpression of circ_0008532 promotes, and silencing of circ_0008532 inhibits the capacity for invasive in BC cells. In addition, circ_0008532 can directly interact with miR-155-5p and miR-330-5p as an miRNA sponge which mediates the expression of the miR-155-5p/miR-330-5p target gene MTGR1 and downstream Notch signaling.

**Conclusions:**

Circ_0008532 may act as an oncogene in BC through a novel circ_0008532/miR-155-5p, miR-330-5p /MTGR1/Notch pathway axis, which in turn may provide potential biomarkers and a therapeutic target for the management of bladder cancer.

## Background

Bladder cancer (BC) is one of the most common malignancies in the genitourinary system, with approximately 400,000 new cases diagnosed annually and over 165,000 deaths [1]. Although treatment such as transurethral resection and intravesical chemotherapy may be successfully applied for non-muscle-invasive bladder cancer (NMIBC), the unfavorable prognosis and high rate of recurrence and metastasis of muscle-invasive bladder cancer (MIBC) result in a 5-year survival rate of approximately 60% [2]. Improved understanding of the mechanisms of BC metastasis and progression will thus likely improve the effectiveness of therapy in patients with advanced stage BC.

Circular RNA (circRNA) are a class of non-coding RNA transcripts that are generated from backsplicing of precursor mRNA [3]. circRNAs are characterized by covalently closed continuous loops without 5′ or 3′ polarities, and are more stable and more resistance to digestion with RNase R than liner transcripts [4]. Studies have reported that circRNAs regulate various biologic processes such as gene expression, transcription, cell proliferation, and apoptosis [5, 6]. In addition, abnormal expression of circRNAs has been found to be involved in the progression of a variety of human cancers [7, 8]. For example, circ-Foxo3 prevents mouse double-minute 2 (MDM2) from inducing Foxo3 ubiquitination and degradation, resulting in increased levels of Foxo3 protein and tumor cell apoptosis [9]. A recent study demonstrated that circ-TTBK2 decreases miR-217 expression and promotes glioma malignancy by regulating the miR-217/HNF1β/Derlin-1 pathway [10]. In bladder cancer, several circRNAs have been shown to act either as a tumor suppressor or an oncogene via different targets [11, 12].

In the present study, we identified a novel circRNA designated circ_0008532 as an oncogene in bladder cancer. Expression of circ_0008532 is significantly upregulated in bladder cancer tissues and cell lines, and is positively associated with bladder cancer progression by sponging miR-155-5p/miR-330-5p to influence the expression of MTGR1 and the activity of Notch signaling. Circ_0008532 may exert regulatory functions and serve as a target for bladder cancer treatment.

## Methods

### Cell culture

Primary cultures of normal bladder urothelial cells (NBUCs) were established from fresh patient specimens. The uroepithelial cell SV-HUC-1 and bladder cancer cell lines (5637, UM-UC-3, TCCSUP, T24, EJ, SCaBER, T24T, J82, SW780) were obtained from the Cell Bank of the Chinese Academy of Sciences (Shanghai, China). All these cell lines were cultured in RPMI 1640 medium supplemented with 10% fetal bovine serum (FBS) (Gibco).

### Tissue specimens

Ten bladder cancer tissues and matched adjacent non-tumor bladder tissues were obtained from the Department of Urology, Huazhong University of Science and Technology affiliated Union Hospital and stored in liquid nitrogen pending use. To select adjacent non-tumor bladder tissues, grossly normal mucosa from the resection margin most distant from tumor was carefully excised and subjected to frozen section evaluation in order to exclude dysplasia and the presence of carcinoma cells. The urothelium and submucosal layers of an adjacent area was then carefully peeled off and placed immediately in liquid nitrogen.

### RNA extraction and quantitative real-time PCR (real-time qPCR)

Total RNA was extracted from cells and fresh tissue using the Trizol (Invitrogen) kit according to the manufacturer’s instructions, and was reverse transcribed using the RevertAid First Strand cDNA SynthesisKit (Thermo Scientific, MA, USA). Subsequently, real-time qPCR was performed on a StepOne Plus real-time PCR system (Life Technologies, Carlsbad, CA). GAPDH was used as an internal control. The sequences of primers are provided in the Additional file 1: Table S1. The 2 − ΔΔCT method was used to calculate relative expression of mRNA.

### Western blotting

Cells and tissue samples were lysed in RIPA lysis buffer, and protein concentrations were determined using a BCA Protein Assay Kit (Thermo Scientific, MA, USA). Cell/tissue lysates were separated with SDS-PAGE gels and transferred to a polyvinylidene difluoride (PVDF) membrane (Millipore, Eschborn, Germany). The blots were blocked in 5% milk for 1 h at room temperature. PVDF membranes were incubated with the primary antibodies overnight in a cold room at 4 °C. Subsequently, bound primary antibodies were reacted with corresponding secondary antibodies for 1 h at room temperature and evaluated with by chemiluminescence.

### Plasmids, lentiviral infection, and transfection

Human circRNA_0008532 and MTGR1 cDNA was amplified by PCR and cloned into a lentiviral vector (GeneChem, Shanghai, China). Oligos of circ_0008532 and MTGR1 shRNAs were synthesized and inserted into a lentiviral vector (GeneChem, Shanghai, China). Stable cell lines were selected for 10 days with 0.5 mg/ml puromycin. The miR-155-5p\miR-330-5p mimics, negative control, and anti-miR-155-5p\anti-miR-330-5p inhibitor were purchased from RiboBio (Guangzhou, China). MiRNA or miRNA inhibitor was transfected with Lipofectamine 2000 reagent (Invitrogen) according to the manufacturer’s instructions.

### Migration and invasion assays

The capacity for cell migration and invasion was evaluated by using Transwell chambers (Corning Life Sciences, MA, USA). After pretreatment, 4 × 10^4^ cells suspended in 200 μL of serum-free medium were seeded into the upper chamber of the Transwell system, and medium supplemented with 10% FBS was added to the lower chamber. For invasion assays, cells were seeded in pre-coated Matrigel Transwell insert chambers. After incubation for 24 h, cells remaining on the top surface were removed, and cells migrated to the lower surface of the membrane were fixed and stained with 0.1% crystal violet.

### Wound healing assay

Cells were seeded in 12-well plates and grown under permissive conditions until 90% confluence was reached. A linear wound was created in the confluent monolayer using a pipette tip, and cells were incubated for 24 h in serum-free medium in a temperature-and CO_2_-controlled incubator. Wound closure was evaluated by measuring the distance between opposite edges of the wound.

### HUVEC tube formation assay

One hundred μl of precooled Matrigel (Becton, Dickinson and Company, NJ, USA) was coated into each well of a 24-well plate and polymerized for half an hour at 37 °C. HUVECs (1 × 10^5^) with 500 μl medium from different groups were added to each well and incubated at 37 °C under 5% CO_2_. Wells were evaluated every 2 h. At the proper time, capillary tube structures were photographed under a bright-field microscope, and quantified by measuring the total length of the completed tubes.

### RNA fluorescence in situ hybridization (RNA-FISH)

Cy3-labeled circ_0008532 and Dig-labeled locked nucleic acid miR-155-5p and miR-330-5p probes were purchased from RiboBio (Guangzhou, China). Images were obtained using a Fluorescent in Situ Hybridization kit (RiboBio, Guangzhou, China) following the manufacturer’s instructions. All data were analyzed with a Nikon A1Si Laser Scanning Confocal Microscope (Nikon Instruments Inc., Japan).

### RNase R digestion

Total RNA (2 μg) was incubated for 20 min at 37 °C with or without 3 U/μg of RNase R. The resulting RNA was purified using an RNeasy MinElute Cleanup Kit (Qiagen, Now York, USA).

### RNA binding protein immunoprecipitation assay (RIP)

The RNA binding protein immunoprecipitation (RIP) assay was performed using the Magna RIP Kit (Millipore, USA) and Ago2 antibody (Cell Signaling Technology, USA) in accordance with the manufacturer’ instructions. In brief, 10^7^ transfected cells were washed in ice-cold PBS twice, lysed in an equal volume of RIP lysis buffer and incubated with 2 μg of primary antibodies for 2 h at 4 °C. Fifty μ of prepared magnetic beads were subsequently added to each sample and incubated at 4 °C overnight. Beads were washed briefly with RIP buffer five times and resuspended in Trizol (Invitrogen). The binding products were detected with real-time qPCR.

### In vivo metastasis assays

All animal experiments were carried out in accordance with NIH Guidelines for the Care and Use of Laboratory Animals and approved by the Animal Care Committee of Tongji Medical College. BALB/c-nu mice (3–4 wks of age) were purchased from the Center of Experimental Animals of Tongji Medical College at Huazhong University of Science and Technology, and were randomly divided into groups (*n* = 5/group). 2 × 10^6^ cells were injected into the tail-vein of each mouse to establish a metastasis model. On day 49, all animals were anesthetized with xylazine (10 mg/kg) and were then euthanized by cervical dislocation. Lungs were excised and subjected to pathologic examination. The In Vivo Optical Imaging System (In Vivo FX PRO, Bruker Corporation) was used to acquire fluorescent images of xenografts in nude mice.

### Statistical analysis

Statistical analysis were conducted with SPSS 16.0 software and significance was analyzed with the Student’s t-test. The cut-point of MTGR1 was defined as the median. Overall survival and recurrence-free survival curves were calculated with the Kaplan–Meier method and compared using the log-rank test. In this study, *P* < 0.05 was considered statistically significant. Data from at least 3 independent experiments are expressed as mean ± SD.

## Results

### Upregulation of circRNA circ_0008532 in BC tissues

**Fig. 1:**
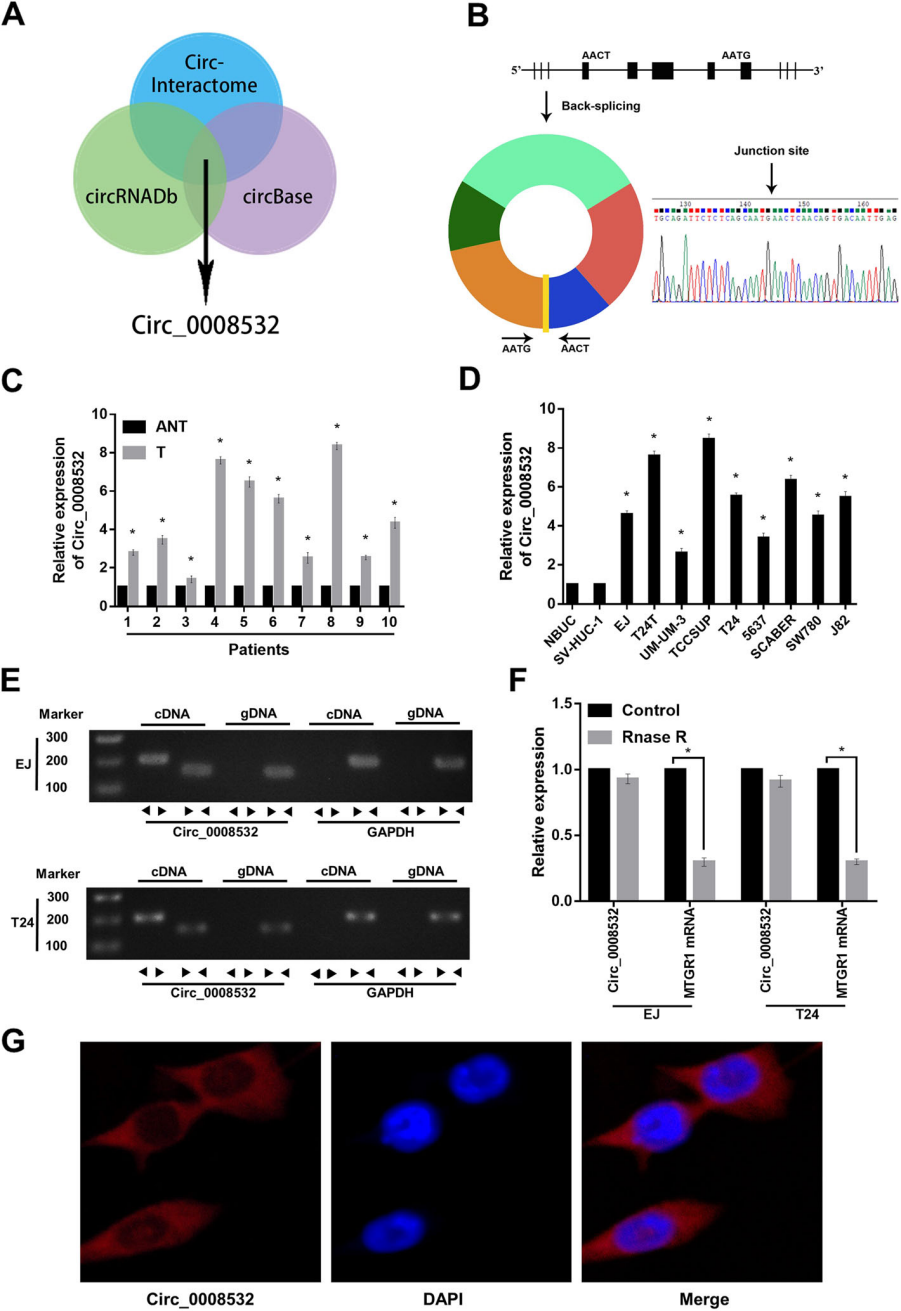
Identification of circ_0008532 in bladder cancer. **a**-**b** The Venn and schematic diagrams show that five exons derived from MTGR1 constitute circ_0008532. The existence of circ_0008532 was demonstrated by PCR and its back-splicing junction was verified by Sanger sequencing. **c** The expression level of Circ_0008532 in 10 pairs of bladder cancer and adjacent non-tumor tissue. **d** The expression level of Circ_0008532 in normal bladder urothelial cells (NBUCs), human uroepithelial cells (SV-HUC-1) and bladder cancer cell lines (EJ, T24T, UM-UC-3, TCCSUP, T24, 5637, SCABER, SW780 and J82). **e** PCR assay with divergent or convergent primers indicating circ_0008532 is present in the EJ and T24 cell lines. GAPDH was used as a negative control. **f** Real-time qPCR analysis of the expression of circ_0008532 after RNase R treatment in EJ or T24 cells. **g** RNA-FISH indicates the location of circ_0008532. Nuclei were stained blue with DAPI. Circ_0008532 is stained red with cy3. Bar graphs show the statistical analysis of three independent experiments (* *P* < 0.05)

Analysis with the circRNA sequencing databases CircInteractome, circBase, and circRNADb revealed that circ_0008532 is derived from MTGR1 (Fig. 1 a). This circular product was amplified with divergent primers and confirmed by Sanger sequencing (Fig. 1 b). We then measured the expression of circ_0008532 in 10 BC tissues and matched adjacent normal tissues using real-time qPCR. The expression of circ_0008532 was markedly upregulated in BC tissues compared with adjacent normal tissues. Moreover, circ_0008532 expression was increased in BC cell lines compared with normal urothelial cells (Fig. 1 c and d). We next designed convergent primers and divergent primers to amplify linear and circular RNA based on cDNA and genomic DNA (gDNA) from cell lines EJ and T24. Circ_0008532 was only amplified by divergent primers in cDNA, and no amplification product was observed in gDNA (Fig. 1 e). By using real-time qPCR, we confirmed that circ_0008532 is resistant to RNase R, while MTGR1 mRNA is significantly reduced after RNase R treatment (Fig. 1 f). We then performed RNA fluorescence in situ hybridization (FISH) assays to identify the subcellular localization of circ_0008532. A Cy3-labeled probe specific for circ_0008532 was used for RNA-FISH. The images indicate that circ_0008532 is mainly localized in a punctate pattern in the cytoplasm (Fig. 1 g). These results demonstrated circ_0008532 was overexpressed in BC tissues and cell lines, and was predominantly localized in the cell cytoplasm.

### Circ_0008532 promotes progression of BC in vitro and in vivo

**Fig. 2:**
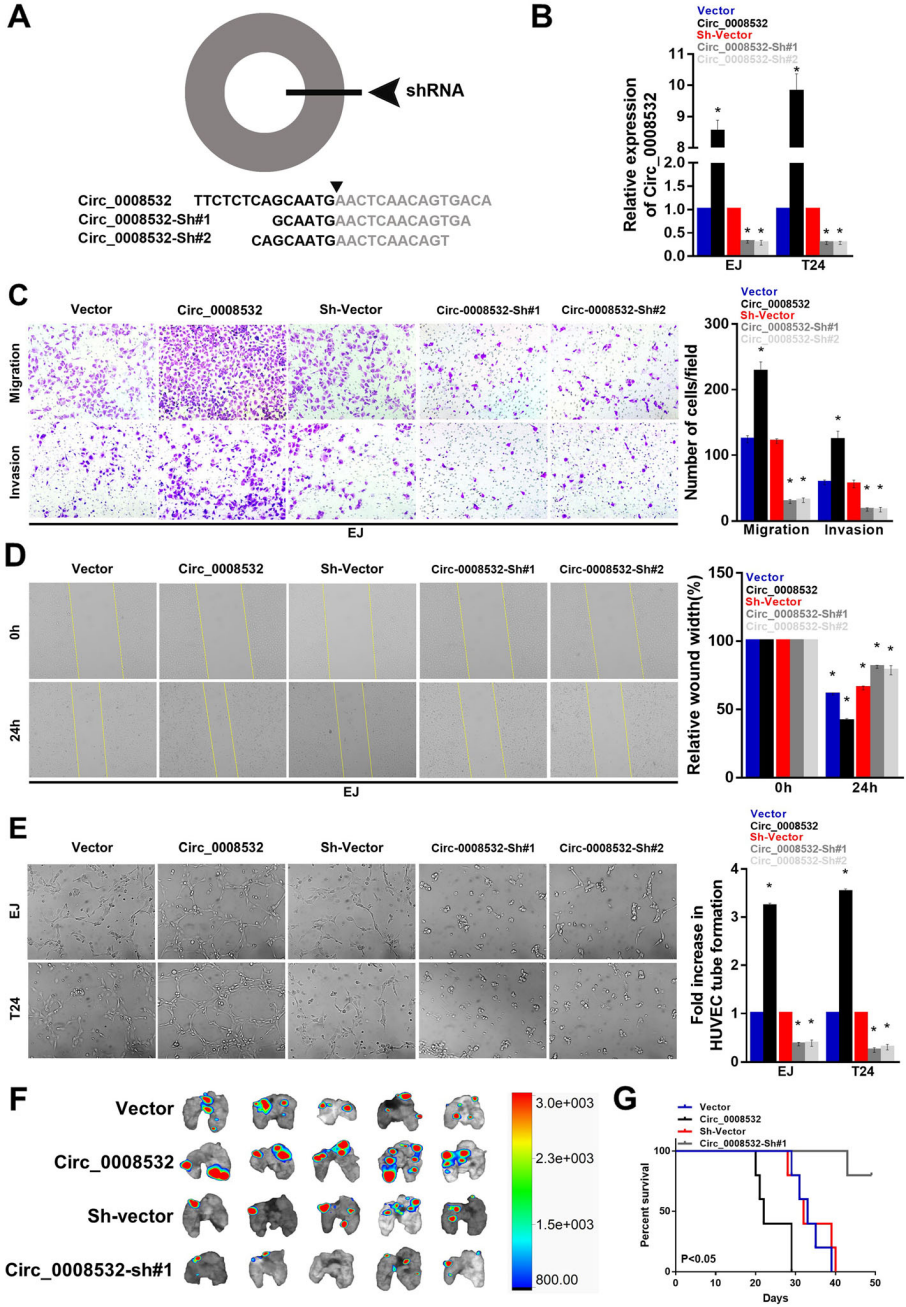
Circ_0008532 promotes BC cell migration, invasion, angiogenesis, and tumor metastasis. **a** Schematic representation of the sites of the siRNA specific to the back-splice junction of circ_0008532. **b** The level of circ_0008532 in EJ and T24 cells with stable expression or silencing of circ_0008532. **c** Representative pictures (left panel) and quantification (right panel) of invaded cells analyzed using a Transwell Matrigel assay. **d** Representative pictures (left panel) and percentage of the original wound area (right panel) of EJ cells analyzed using a wound-healing assay. **e** Representative images (left panel) and quantification (right panel) of HUVECs cultured on Matrigel-coated plates with conditioned medium from EJ cells. **f** Representative image of metastatic lung colonization in nude mice . **g** Kaplan–Meier curves for nude mice. Bar graphs show the statistical analysis of three independent experiments (* *P* < 0.05)

To investigate the effect of circ_0008532 on cell migration, invasion, and angiogenesis, circ_0008532-overexpressing (circ_0008532) and circ_0008532-knockdown (circ_0008532-sh#1 and circ_0008532-sh#2) T24 and EJ cell lines were established. The Transwell assay showed that cells overexpressing circ_0008532-exhibited a significantly increased capacity for migration and invasion, whereas circ_0008532-knockdown cells had a decreased capacity (Fig. 2 c). In addition, wound healing assays showed similar results that cells with high circ_0008532 expression exhibit high mobility (Fig. 2 d). The HUVEC tube formation assay showed that circ_0008532-ovexpressing cells exhibit increased ability for induction of tubule formation by human umbilical vein endothelial cells (Fig. 2 e). Moreover, athymic nude mice treated with tail vein injection of EJ cells stably transfected with circ_0008532 displayed more metastatic pulmonary colonies and worse survival (Fig. 2 f-g). These results suggest that circ_0008532 promotes bladder cancer metastasis and aggressiveness.

### Circ_0008532 acts as a sponge for miR-155-5p/miR-330-5p which is downregulated in bladder cancer

**Fig. 3:**
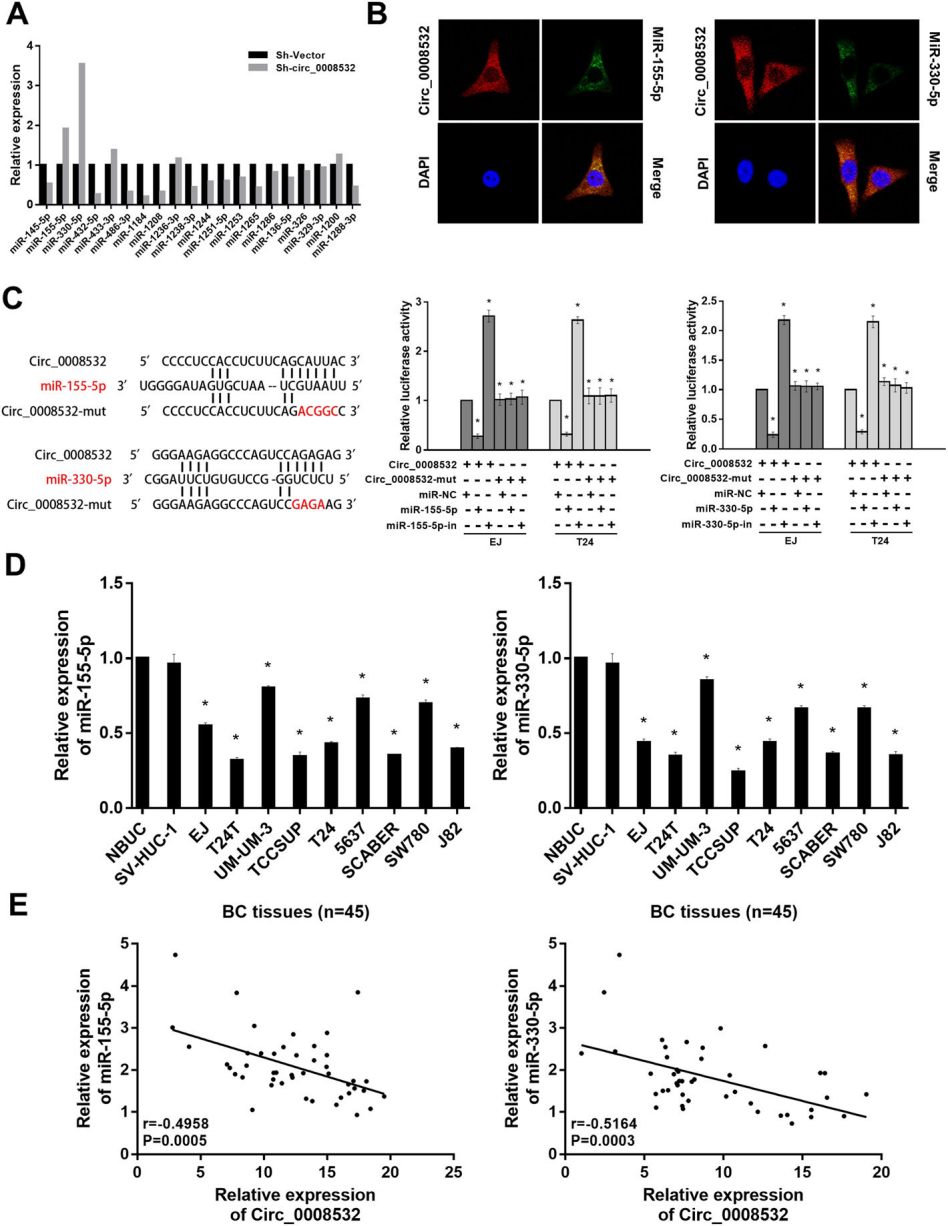
Circ_0008532 acts as a sponge for miR-155-5p/miR-330-5p. **a** MiR-155-5p and miR-330-5p were pulled down with anti-ago2 antibody more effectively in the circ_0008532 knock-down group than in the control group. **b** RNA-FISH revealed that circ_0008532 and miR-155-5p/miR-330-5p colocalizes in the cytoplasm in EJ cells. Nuclei were stained blue with DAPI. Circ_0008532 was stained red with cy3. Locked nucleic acid miR-155-5p/miR-330-5p probes were labeled with Dig. **c** Schematic showing the predicted binding sites for circ_0008532 and miR-155-5p/miR-330-5p, and mutation of potential miRNAs-binding sequence in circ_0008532 (left). Luciferase activity in bladder cancer cells cotransfected with a luciferase reporter containing either circ_0008532-wt or circ_0008532-mut and miR-155-5p/miR-330-5p mimics or inhibitors (right). **d** Levels of miR-155-5p/miR-330-5p were downregulated in bladder cancer cells. **e** Correlation of miR-155-5p/miR-330-5p and circ_0008532 expression in 45 BC tissues. Bar graphs show the statistical analysis of three independent experiments (* *P* < 0.05)

Twenty miRNAs were predicted to be potential targets of circ_0008532 through use of CircInteractome. The results of the ago2-RIP assay showed that among these candidate miRNAs, miR-155-5p and miR-330-5p were pulled down with anti-ago2 antibody more effectively in the circ_0008532 knock-down group than in the control group (Fig. 3 a). The RNA FISH assay revealed circ_0008532 and miR-155-5p/miR-330-5p colocalize in the cytoplasm (Fig. 3 b). In addition, the luciferase assay demonstrated that miR-155-5p and miR-330-5p were directly targeted by circ_0008532 (Fig. 3 c). In keeping with these results, the levels of miR-155-5p and miR-330-5p were significantly downregulated in BC cell lines (Fig. 3) and were negatively related by expression of circ_0008532 (Fig. 3 e). These results raise the possibility that circ_0008532 functions as a ceRNA for miR-155-5p and miR-330-5p in bladder cancer cells.

### MiR-155-5p/miR-330-5p inhibits cell migration, invasion, as well as the capacity for angiogenesis and can remedy the positive effects of circ_0008532

**Fig. 4:**
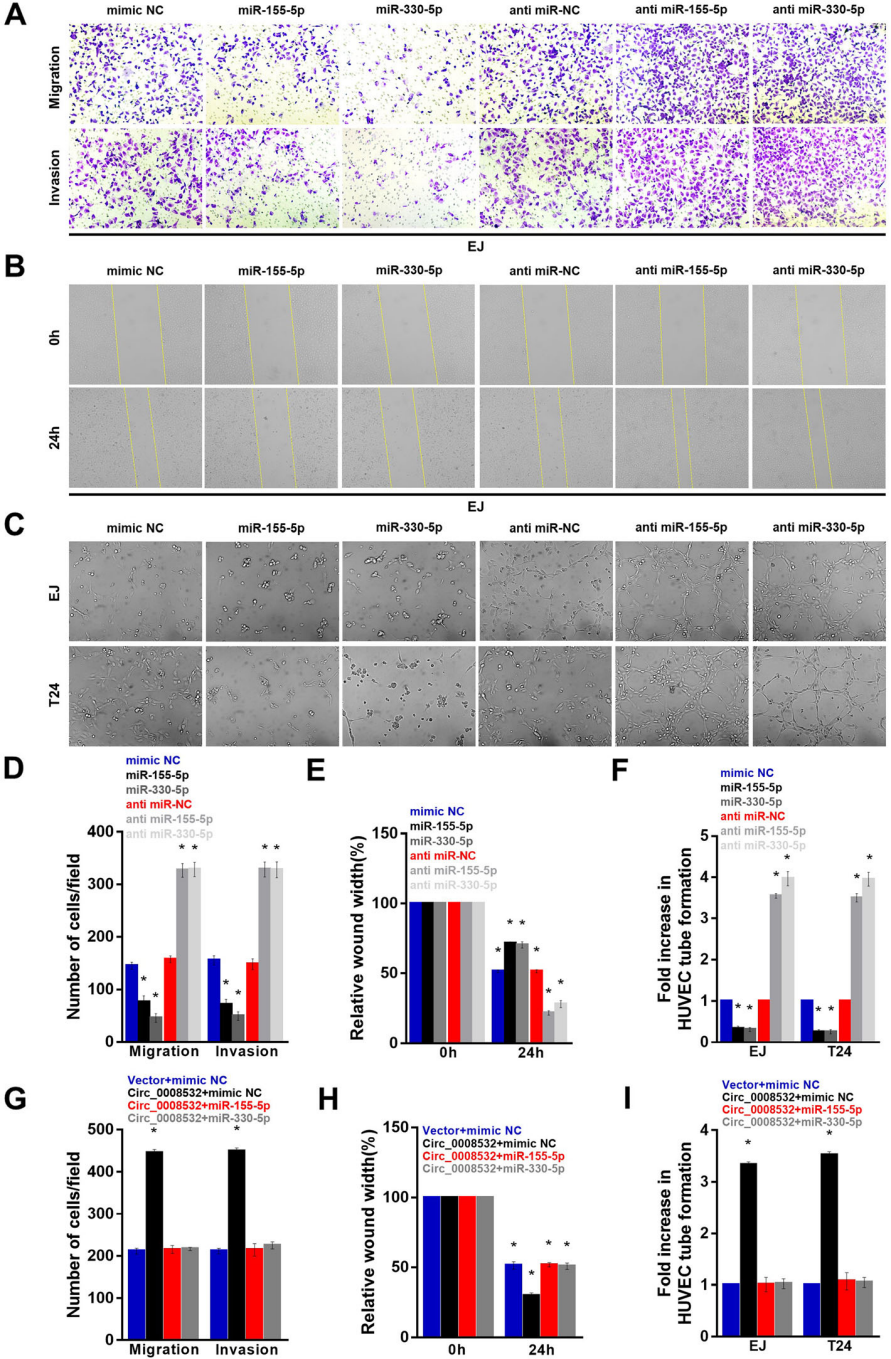
MiR-155-5p/miR-330-5p inhibits cell invasiveness and can remedy the positive effects of circ_0008532. Representative images (**a**) and quantification (**d**) of invaded cells were analyzed using a Transwell Matrigel assay. Representative images (**b**) and percentage of the original wound area (**e**) of EJ cells were analyzed using a wound-healing assay. Representative images (**c**) and quantification (**f**) of HUVECs cultured on Matrigel-coated plates with conditioned medium from EJ cells. (G-I) MiR-155-5p/miR-330-5p counteracts tumor cell invasion (**g**), migration (**h**), and angiogenesis (**i**) induced by circ_0008532. Data are represented as mean ± SD of three independent experiments (* *P* < 0.05)

We next assessed the potential functional roles of miR-155-5p and miR-330-5p in bladder cancer. Enforced expression of miR-155-5p and miR-330-5p significantly inhibited migration, invasion, and angiogenesis of EJ and T24 cells. In contrast, transfection of miR-155-5p/miR-330-5p inhibitor had the opposite effect (Fig. 4 a-f). Moreover, miR-155-5p/miR-330-5p overexpression effectively abolished the tumor-promoting effect of circ_0008532 in BC (Fig. 4 g-i).

### MiR-155-5p/miR-330-5p directly targets MTGR1

**Fig. 5:**
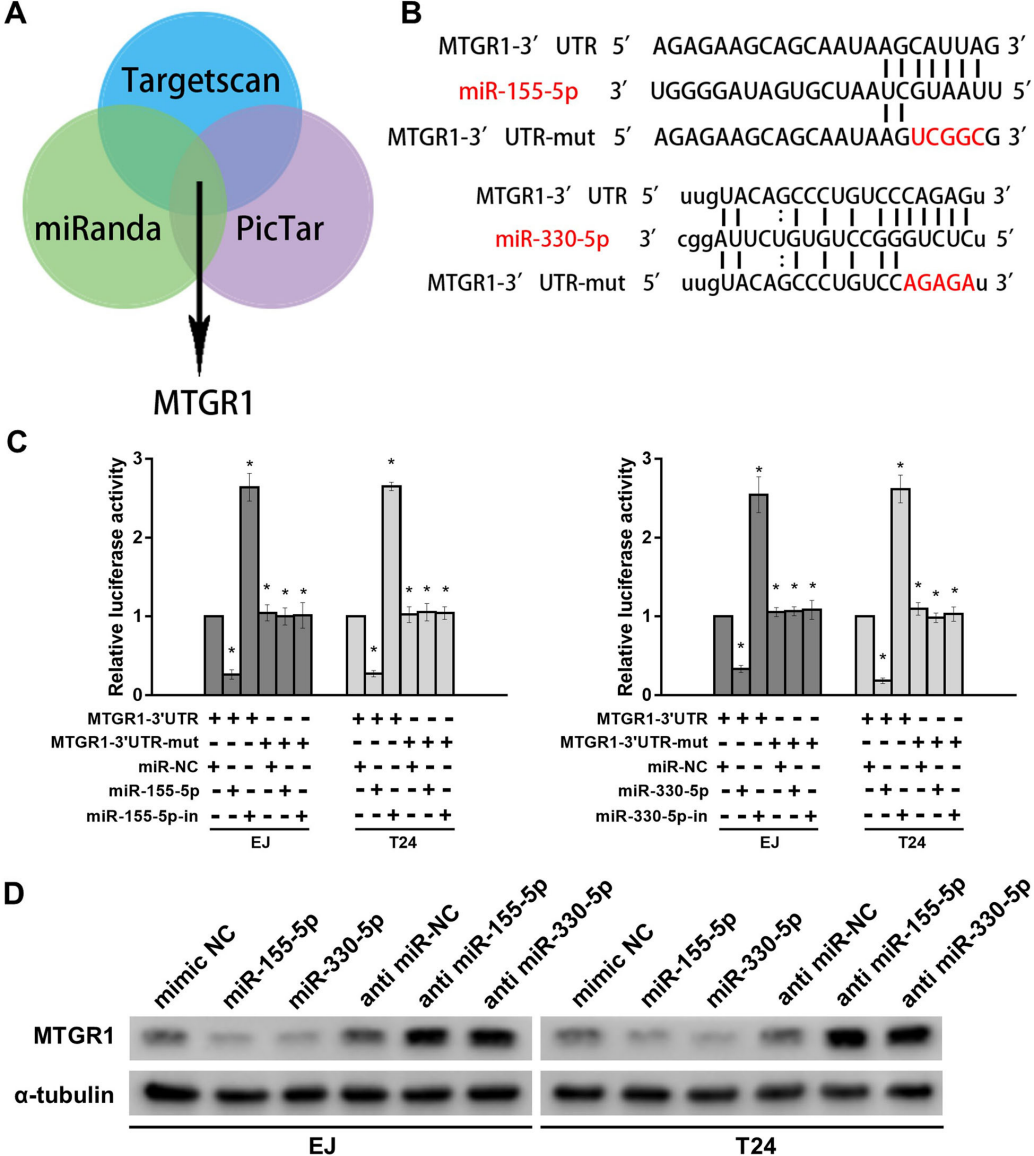
MiR-155-5p/miR-330-5p directly targets MTGR1. **a** The Venn diagram shows MTGR1 is commonly predicted by TargetScan, miRanda and PicTar. **b** The binding sites of MTGR1 and miR-155-5p/miR-330-5p. **c** Luciferase activity in bladder cancer cells cotransfected with a luciferase reporter containing either MTGR1 3’UTR-wt or MTGR1 3’UTR -mut and miR-155-5p/miR-330-5p mimics or inhibitors. **d** The protein levels of MTGR1 detected by western-blotting in miR-155-5p/miR-330-5p overexpressing and knock-down cells. Bar graphs show the statistical analysis of three independent experiments (* *P* < 0.05)

In this in silico study using 3 bioinformatics algorithms including Targetscan, miRanda and PicTar, we found that MTGR1 may be a target of miR-155-5p and miR-330-5p (Fig. 5 a). Using the luciferase assay, we found that miR-155-5p and miR-330-5p do in fact directly target MTGR1 (Fig. 5 b and c). Western blotting assays showed that MTGR1 was significantly suppressed by upregulating miR-155-5p and miR-330-5p expression levels (Fig. 5 d).

### MTGR1 is upregulated in BC and promotes BC cell migration, invasion, and angiogenesis

**Fig. 6:**
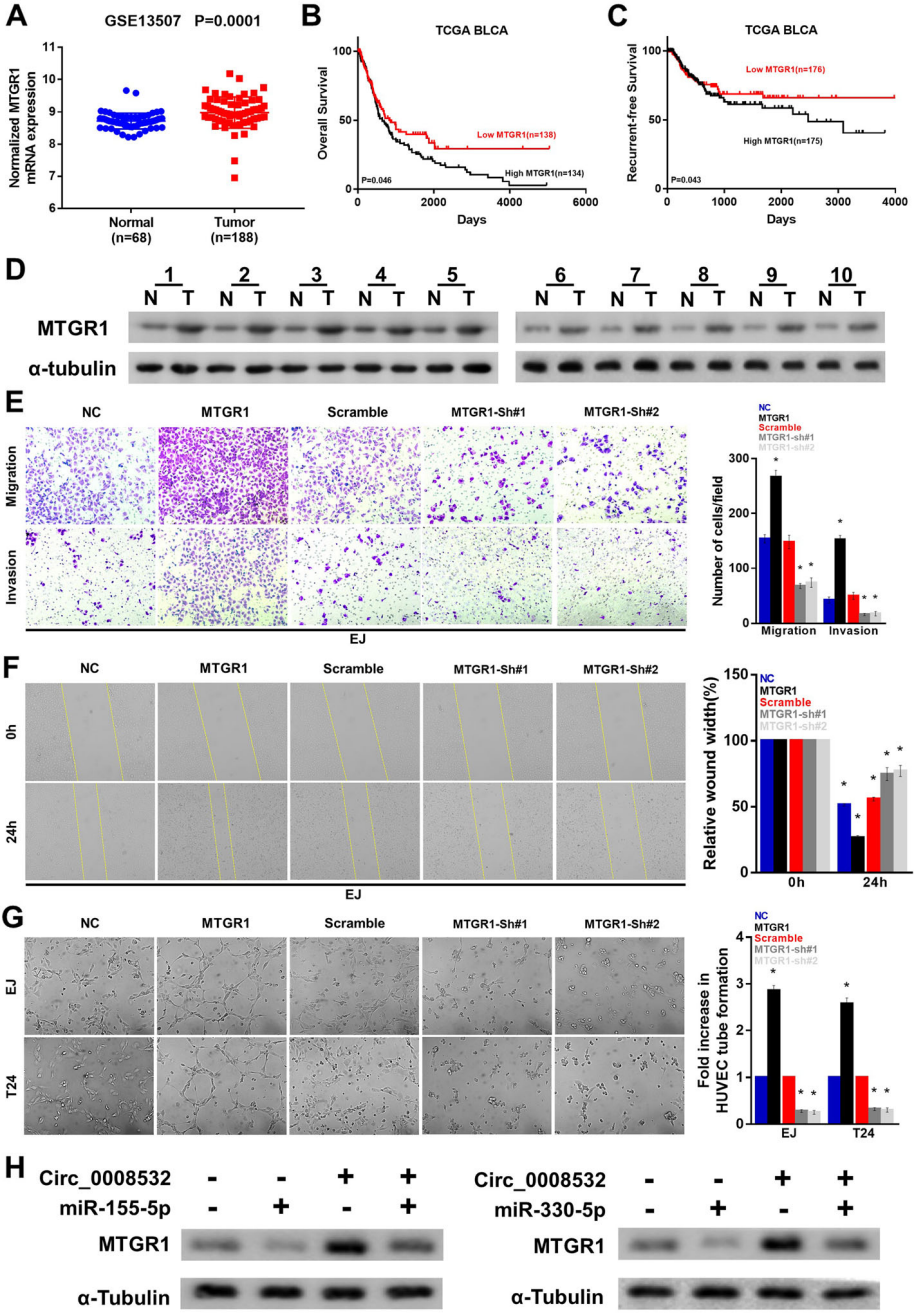
MTGR1 is upregulated in BC and promotes BC cell migration, invasion, and angiogenesis. **a** MTGR1 expression in GSE13507 dataset. (**b**-**c**) Overall and recurrence-free survival in the TCGA BLCA dataset with low versus high levels of NUDT21 mRNA. **d** MTGR1 protein expression in 10 bladder cancers (T) and paired adjacent non-tumor tissues (N). **e** Representative pictures (left panel) and quantification (right panel) of cells which have invaded were analyzed using a Transwell Matrigel assay. **f** Representative pictures (left panel) and percentage of the original wound area (right panel) of EJ cells were analyzed using a wound-healing assay. **g** Representative images (left panel) and quantification (right panel) of HUVECs cultured on Matrigel-coated plates with conditioned medium from EJ cells. (H) Circ_0008532 and miR-155-5p/miR-330-5p co-regulate the protein level of MTGR1

Moreover, expression data downloaded from our bioinformatics platform showed that MTGR1 is significantly upregulated in BC tissues compared with normal tissues (Fig. 6 a), and that lower expression of MTGR1 is correlated with better prognosis (Fig. 6 b and c). Our experiments also showed that MTGR1 is upregulated in BC tissues compared with that in matched non-cancerous tissues (Fig. 6 d), and that MTGR1 may promote BC cell migration, invasion, and angiogenesis (Fig. 6 e-g). Furthermore, western blotting assays showed that overexpression of miR-155-5p/miR-330-5p partly reverses the elevated expression of MTGR1 induced by circ_0008532 overexpression in BC cells (Fig. 6 h).

Circ_0008532 improves cell migration, invasion, and angiogenesis by suppressing the Notch signaling pathway.

**Fig. 7:**
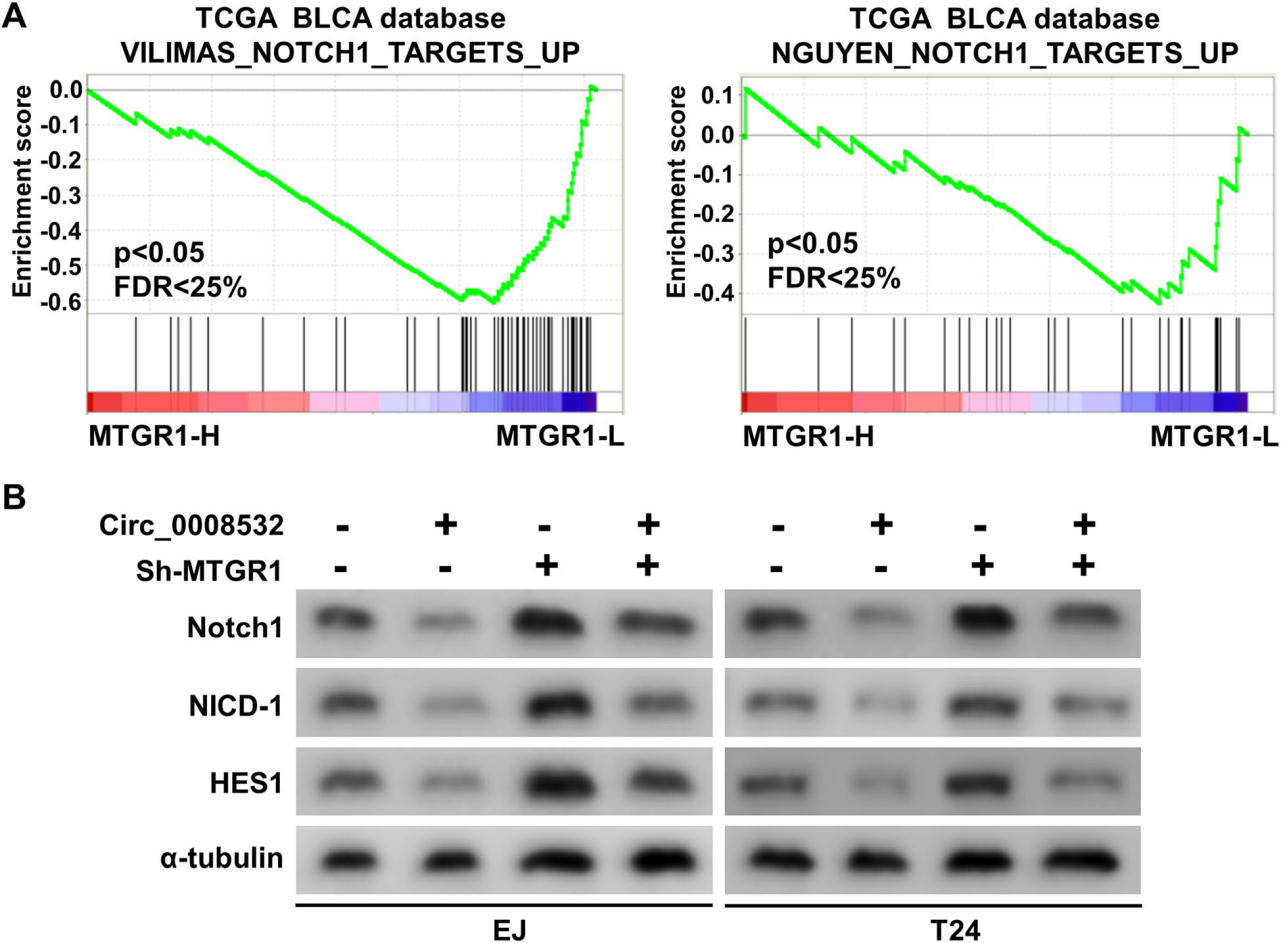
Circ_0008532 suppresses Notch signaling pathway. **a** GSEA plot showing that MTGR1 expression is negatively correlated with Notch signaling. **b** Western blotting analysis of Notch1, NICD-1 and HES1 expression in cells as indicated. α-Tubulin is used as a loading control

To further elucidate the molecular mechanism of circ_0008532 regulated BC progression, Gene Set Enrichment Analysis (GSEA) was performed based on mRNA expression data from the TCGA, and results indicated that MTGR1 overexpression is negatively associated with the activation of Notch signaling (Fig. 7 a). To further determine the involvement of circ_0008532 in the Notch signaling pathway, we examined the expression of downstream genes of Notch. Western blotting revealed that expression of Notch1, NICD-1 and HES1 were remarkably decreased in circ_0008532-overexpressing cells. Moreover this result was reversible by knock down of MTGR1expression of (Fig. 7 b). These results suggest circ_0008532 may suppress the activation of Notch signaling by regulating MTGR1 expression.

## Discussion

Muscle invasive bladder cancer (BC) is a deadly disease. Currently, the therapeutic approach to BC is based primarily on surgery and standard chemotherapy, and novel therapeutic strategies are urgently needed for the treatment of metastatic BC [13]. In recent years, the role of dysregulated non-coding RNAs (ncRNAs) in the proliferation, migration, invasion, and angiogenesis of cancer cells have generated significant scientific interest. The non-coding part of the genome accounts for more than 90% of the human genome. Studies have demonstrated that ncRNAs play critical roles in tumorigenesis and pathologic processes in many human cancers [14, 15]. CircRNA, as abundant stable ncRNAs, has been demonstrated to play a prominent upstream role in BC development. For example, cTFRC is upregulated in BC, and is involved in invasion and proliferation in BC cell lines [16]. Moreover, circRNA hsa_circ_0068871 regulates FGFR3 expression and activates STAT3 by targeting miR-181a-5p to promote bladder cancer progression, showing some of the important roles of circRNA in BC [17].

In the current study, we explored the effect of the novel circRNA circ_0008532 on the aggressiveness of BC and identified the regulatory mechanism of miR-155-5p/miR-330-5p/MTGR1 signaling. Our results indicate that elevated expression of circ_0008532 increases the invasive capacity of BC cells. Circ_0008532 functions as a molecular sponge for miR-155-5p and miR-330-5p and weakens the inhibitory effect of these molecules on the downstream target gene MTGR1. These results suggest that Circ_0008532 has the potential to regulate the migration, invasion, and angiogenesis of BC cells, which in turn promote BC progression.

It is well-known that circRNAs may act as miRNA sponges to regulate the expression of target genes [18]. For example, Circ-EZH2 promotes cell growth, migration and invasion but inhibits cell apoptosis through miR-1265 Sponge Activity in Glioma [19]. CircARHGAP10 suppresses cell proliferation and metastasis in non-small-cell lung cancer by acting as a miR-150-5p sponge which promotes GLUT1 expression [20]. Here we found that circ_0008532 enhances the aggressiveness of BC mainly through sponging miR-155-5p and miR-330-5p. Circ_0008532 is located mainly in the cytoplasm, which is regarded as a major characteristic of miRNA sponges. Second, the circ_0008532 expression level is negatively correlated with miRNAs expression. Third, bioinformatics prediction and luciferase reporter assays show that circ_0008532 and the MTGR1 3′ UTR share identical miR-155-5p/miR-330-5p response elements and may therefore bind competitively to miR-155-5p/miR-330-5p. This study thus reveals that a circ_0008532/miR-155-5p/miR-330-5p/MTGR1 axis exists in BC.

MTGR1, also known as CBFA2T2, is a member of the Myeloid Translocation Gene (MTG) family, and this family of molecules are transcriptional corepressors lacking both enzymatic activity and DNA binding capabilities [21]. The MTGR1 gene is located at chromosome 20q11.21 and has been found to be involved in the regulation of the nuclear corepressor/histone deacetylase complex in hematopoietic differentiation [22]. In addition, MTGR1 has been shown to contribute to Notch signaling inhibition and regulation of intestinal lineage allocation [23]. Evidence has demonstrated that MTGR1 is involved in the development of cancer. Barrett et al. reported that MTGR1 is required for efficient inflammatory carcinogenesis in the murine AOM/DSS colitis-associated carcinoma model [24]. Parang et al. showed that MTGR1 is downregulated in human colorectal cancer and has a context-dependent effect on intestinal tumorigenesis [25]. However, the biologic functions of MTGR1 in bladder cancer have not been investigated. Our study shows that MTGR1 is increased in bladder cancer tissues, and that overexpression of MTGR1 leads to enhanced progression in bladder cancer. Furthermore, circ_0008532 promotes progression of bladder cancer by promoting MTGR1 expression and, in turn, inhibits the activity of Notch signaling. Our findings are supported by the study of Rampias and colleagues, who showed that genetic inactivation of Notch signaling leads to tumorigenesis in urothelial cancer [26]. Similarly, Maraver et al. demonstrated that Notch serves as a tumor suppressor in the bladder and that loss of this pathway promotes mesenchymal changes and invasive features [27]. Together, these findings reveal a crucial link between MTGR1 and the Notch signaling pathway during bladder cancer development and progression.

## Conclusions

In conclusion, the current study shows circ_0008532 may be required for BC progression, and this molecule has potential as a clinical biomarker for BC. Moreover, circ_0008532 serves as a sponge for miR-155-5p/miR-330-5p to reduce the inhibitory effect on MTGR1, and thus enhances the expression of MTGR1 and inhibits the activity of the downstream Notch signaling pathway.

## Supplementary information

**Additional file 1: Table S1.** The primer sequence used in this study.

## Data Availability

The datasets used and/or analyzed during the current study are available from the corresponding author on reasonable request.

## Abbreviations

3′-UTR: 3′ Untranslated region
CCK-8: Cell counting KIT-8
circRNA: Circular RNA
FBS: Fetal bovine serum
H&E: Hematoxylin and eosin
IHC: Immunohistochemistry
miRNA: microRNA
qRT-PCR: Quantitative real-time polymerase chain reaction
siRNA: Small interfering RNA
GSEA: Gene set enrichment analysis
TCGA: The cancer genome altas
